# Chemical composition of cassava-based feed ingredients from South-East Asia

**DOI:** 10.5713/ab.22.0360

**Published:** 2023-02-23

**Authors:** Natalia S. Fanelli, Leidy J. Torres-Mendoza, Jerubella J. Abelilla, Hans H. Stein

**Affiliations:** 1 Department of Animal Sciences, University of Illinois, Urbana, IL 61801, USA; 2 DSM Nutritional Products, Mapletree Business City 117440, Singapore

**Keywords:** Animal Feeding, Cassava Products, Chemical Composition, Feed Ingredient, South-East Asia

## Abstract

**Objective:**

Information about the chemical composition of cassava-based feed ingredients is needed to accurately formulate animal diets. A study was conducted to determine the chemical composition of cassava-based feed ingredients and to test the hypothesis that there is variation in chemical composition among cassava products originating from different South-East Asian countries.

**Methods:**

Sources of dried peeled and unpeeled cassava roots, cassava chips, cassava meal, high-ash cassava meal, and cassava residue were used. All samples were analyzed for dry matter, gross energy, nitrogen, amino acids (AA), acid-hydrolyzed ether extract (AEE), ash, minerals, total starch, insoluble dietary fiber, and soluble dietary fiber. Samples of peeled and unpeeled cassava roots, cassava chips, and cassava meal were also analyzed for sugars.

**Results:**

High-ash cassava meal had greater (p<0.05) dry matter and ash, but lower (p<0.05) total starch and gross energy than all other cassava products. Peeled cassava roots, unpeeled cassava roots, and cassava chips had greater (p<0.05) total starch than the other cassava-based ingredients. Cassava residue had greater (p<0.05) concentrations of lysine, insoluble dietary fiber, and soluble dietary fiber compared with the other cassava products, but tryptophan and glutamic acid were greater (p<0.05) in peeled cassava roots, cassava chips, and cassava meal samples compared with the other ingredients. Concentration of most minerals was greater (p<0.05) in high-ash cassava meal than in the other cassava products.

**Conclusion:**

Cassava-based ingredients sold as peeled roots, unpeeled roots, chips, or meal have chemical compositions that are not different from each other, and peeling has little impact on chemical composition. High-ash cassava meal has lower nutritional quality compared with other cassava products due to low starch and gross energy. The high fiber content in cassava residue makes this ingredient more suitable for ruminants and sows than for younger pigs or poultry.

## INTRODUCTION

In most countries in South-East Asia, cassava is mostly used for human consumption, but cassava is also used for starch processing or animal feed [1]. Approximately 60% of cassava produced in South-East Asia takes place in Thailand and Indonesia [2]. Cassava may be used domestically, but cassava grown in Thailand and Vietnam is mainly destined for export or animal feeding [1,2]. Other cassava-based ingredients, such as cassava leaf or silage, have also been used for livestock feeding [1]. Cassava-based feeding programs in South-East Asia are managed based on competition with other energy sources, changes in mechanization procedures, and price fluctuations. As a consequence, cassava production in South-East Asia is expected to increase in the coming years as corn prices are expected to remain elevated [2].

Cassava is a starchy tuber crop grown in tropical and sub-tropical areas and is also known as manioc, tapioca, or yucca [3,4]. In fresh, ensiled, or dried forms, cassava provides energy to animal diets [5]. Cassava cultivation originated in South America, but the crop was later transported to Africa and Asia [1]. In South-East Asia, cassava was first cultivated as a food crop in Indonesia and the Philippines, and later in Thailand [1]. Fresh cassava roots contain 60% to 65% water and must be dried to be preserved [3]. The main concern associated with feeding fresh roots is the presence of cyanogenic glucosides that are toxic to animals when converted to hydrocyanic acid by the intestinal microflora [3,5]. However, processing such as peeling, washing, drying, grinding, steaming, ensiling, cooking, or pelleting may dissipate most of the hydrocyanic acid [6].

Peeled cassava is obtained after the tubers have been water-cleaned and mechanically peeled [7]. Cassava chips are chopped cassava roots that have been naturally dried (sun-dried) or artificially dried and can be produced in a variety of shapes and sizes depending on the slicing and drying methods [1]. Cassava chips may be sold directly, ground into cassava meal, or pelleted [5]. The main component of cassava chips and cassava meal is starch [3]. The solid fibrous residue of cassava is known as cassava residue and represents the remaining residue after most of the starch has been extracted to produce cassava flour [7]. This residue may also be used as an animal feed ingredient [1].

Most studies on the nutrient composition of cassava used only one co-product or are focused on specific nutrients, and there is limited information about the full chemical composition of cassava-based feed ingredients. To fully understand the chemical composition of a feed ingredient it is important to analyze components that add to 100% and such information is not available for cassava-based ingredients. In addition, information about differences in composition among countries in South-East Asia is scarce [8,9]. There is, therefore, a need to characterize these ingredients and provide information about their chemical composition. The objectives of this study were to determine the chemical composition of different cassava-based feed ingredients from South-East Asia and to test the hypothesis that there is variation in chemical composition among cassava co-products.

## MATERIALS AND METHODS

### Description of samples

Cassava-based ingredient samples obtained from South-East Asian cassava suppliers were delivered to DSM Nutritional Products in Singapore. The suppliers provided between 100 and 300 grams of each ingredient. Samples were labeled, cataloged, and then shipped to the University of Illinois, Urbana, IL, USA, where most of the chemical analyses were conducted. There were three samples of dried peeled cassava roots from the Philippines and Thailand, three samples of dried unpeeled cassava roots from the Philippines and Vietnam, six samples of cassava chip products from Thailand and Vietnam, two samples of cassava meal from Thailand, two samples of high-ash cassava meal from Indonesia, and six samples of cassava residue products from Indonesia and Vietnam. High-ash cassava meal samples were described by suppliers as cassava meal, but due to the high mineral concentration, they were renamed high-ash cassava meal.

### Chemical analysis

Samples of all cassava-based ingredients were finely ground and analyzed for dry matter (method 930.15) [10] and ash (method 942.05) [10], and gross energy was analyzed using an isoperibol bomb calorimeter (model 6400; Parr Instruments, Moline, IL, USA). Samples were analyzed for amino acids (AA [method 982.30 E a, b, and c]) [10] on a Hitachi AA Analyzer (model L8800; Hitachi High Technologies America Inc., Pleasanton, CA, USA) and nitrogen was analyzed by combustion (method 990.03) [10] using a LECO FP628 Nitrogen Analyzer (LECO Corp., Saint Joseph, MI, USA). Crude protein was calculated as nitrogen×6.25. Acid-hydrolyzed ether extract (AEE) was analyzed using 3N HCl (method 2003.06; Ankom^HCl^; Ankom Technology, Macedon, NY, USA) followed by crude fat extraction using petroleum ether (Ankom^XT15^; Ankom Technology, USA). Insoluble and soluble dietary fiber were quantified according to method 991.43 [10] using the Ankom^TDF^ Dietary Fiber Analyzer (Ankom Technology, USA). Total dietary fiber was calculated as the sum of insoluble and soluble dietary fiber. Total starch was analyzed using the glucoamylase procedure (method 979.10) [10]. Minerals were also analyzed (method 985.01 a, b, and c) [10] using inductively coupled plasma-optical emission spectrometry (ICP-OES; Avio 200; PerkinElmer, Waltham, MA, USA). Sample preparation included dry ashing at 600°C for 4 h (method 942.05; 10) [10] and wet digestion with nitric acid (method 3050 B) [11]. Sugars and oligosaccharides including glucose, fructose, maltose, sucrose, stachyose, and raffinose were analyzed in peeled cassava roots, unpeeled cassava roots, cassava chips, and cassava meal samples using high-performance liquid chromatography (Dionex App Notes 21 and 92). All analysis were performed on duplicate samples of each ingredient.

### Calculations and statistical analysis

For peeled cassava roots, unpeeled cassava roots, cassava chips, and cassava meal, analyzed proximate components were added and subtracted from the concentration of dry matter in each sample to calculate the rest fraction according to the following equation:


$$\begin{array}{ll} \text{Rest fracion}=[\; & \text{Dry matter}-(\text{crude protein}\\ & +\text{AEE}+\text{ash}+\text{total dietary fiber}\\ & +\text{total starch}+\text{glucose}+\text{fructose}\\ & +\text{maltose}+\text{sucrose}+\text{stachyose}\\ & +\text{raffinose})]. \end{array}$$

The rest fraction for high-ash cassava meal and cassava residue samples was calculated using the same equation, with the exception that sugars were not analyzed in these samples and, therefore, not included in the equation.

The coefficient of variation and average of all samples from each country within each group of feed ingredient were calculated if there were more than one sample per country. Normality of residues was verified using the UNIVARIATE procedure (SAS 9.4 Institute Inc. Cary, NC, USA). Data were analyzed by analysis of variance (ANOVA) using the PROC MIXED procedure in SAS to test statistical differences among ingredients. To determine homoscedasticity, the Levene’s test was used. The BOXCOX procedure was then applied to determine the optimal lambda for data transformation. ANOVA was performed on transformed data and assumptions were met. However, when transformation was not possible, the SATTERTHWAITE function in the MIXED procedure was used to allow results to be adjusted for unequal variances and to provide an alternate p-value when variances differed. The replicate sample was the experimental unit for all analyses. The feed ingredient was the fixed effect, and the replicate sample was the random effect. Means were calculated using the LSMEANS statement in SAS, and when significant, means were separated using the PDIFF option in the MIXED procedure. Results were considered significant at p<0.05.

## RESULTS

### Peeled and unpeeled cassava roots

Dried peeled cassava roots from the Philippines and Thailand had a chemical composition that was not different, with less than 30% coefficient of variation for most nutrients (Table 1). Likewise, the chemical composition of dried unpeeled cassava roots from the Philippines and Vietnam was not different, with less than 30% coefficient of variation for most nutrients (Table 2). The main nutrients in peeled and unpeeled cassava root samples were starch and total dietary fiber. These samples also contained an average of 2% crude protein, 0.7% AEE, 1.4% sugars, and 1% minerals. The average rest fraction in peeled cassava roots was 6.9%, whereas the average rest fraction in unpeeled cassava roots was 3.3%.

### Cassava chips, cassava meal, and cassava residue

The nutrient composition of cassava chips from Thailand was not different from that of chips from Vietnam (Table 3). With the exception of soluble dietary fiber and most sugars, the coefficient of variation for the analyzed components of cassava chips from Vietnam was low accounting for less than 30%. The main nutrients in cassava chips were starch and total dietary fiber. These samples also contained an average 2.5% crude protein, 0.7% AEE, 2% sugars, and 1% minerals. Sucrose was the sugar present in greatest concentration in most cassava chip samples, particularly those from Vietnam. The average rest fraction in cassava chips was 5.7%.

The main nutrients in cassava meal from Thailand were starch, total dietary fiber, and ash. The samples contained an average of 2.7% crude protein, 0.8% AEE, 1.7% sugars, and 1.2% minerals (Table 4). The average rest fraction in cassava meal was 5.3%. High-ash cassava meal samples from Indonesia contained approximately 2% crude protein, 0.6% AEE, 38% ash, and 23% total dietary fiber (Table 5). These samples also contained approximately 30% starch. The average rest fraction in high-ash cassava meal accounted for less than 1%.

Cassava residue samples from Indonesia, Thailand, and Vietnam had on average of 35% total dietary fiber, 2.3% crude protein, 1.4% AEE, and 1.2% minerals (Table 6). These samples also contained approximately 45% starch. The coefficient of variation for most nutrients in cassava residue from Thailand and Vietnam was less than 30%. The average rest fraction in cassava residue samples accounted for less than 1%.

### Comparison of cassava-based feed ingredients

High-ash cassava meal had greater dry matter and ash (p = 0.044 and p<0.001, respectively), but lower (p<0.001) gross energy and total starch compared with the other cassava ingredients (Table 7). Cassava meal had greater (p<0.001) concentration of ash than peeled cassava roots and cassava chips. Total starch was greater (p<0.001) in peeled cassava roots, unpeeled cassava roots, and cassava chips compared with the other cassava co-products. Insoluble, soluble, and total dietary fiber were greater (p<0.001) in cassava residue samples than in all other cassava ingredients, however, total dietary fiber in high-ash cassava meal was not different from that of cassava residue. There were no differences in crude protein and AEE among cassava co-products. There were also no differences in the concentration of sugars and oligosaccharides among peeled cassava roots, unpeeled cassava roots, cassava chips, and cassava meal.

No differences in the concentration of most AA among cassava co-products were observed. However, lysine was greater (p = 0.004) in cassava residue compared with peeled cassava roots, unpeeled cassava roots, cassava chips, and high-ash cassava meal. The concentration of tryptophan was greater (p<0.001) in peeled cassava roots, cassava chips, and cassava meal than in unpeeled cassava roots, high-ash cassava meal, and cassava residue. Likewise, glutamic acid was greater (p = 0.001) in peeled cassava roots, cassava chips, and cassava meal than in high-ash cassava meal and cassava residue.

The high-ash cassava meal had greater (p <0.001) concentration of calcium, magnesium, potassium, sulfur, cooper, iron, manganese, and zinc compared with the other cassava co-products. Cassava residue had greater (p<0.001) concentration of calcium and iron than peeled cassava roots, unpeeled cassava roots, and cassava chips. Likewise, cassava meal had greater (p<0.001) concentration of manganese and greater (p = 0.004) concentration of iron compared with peeled cassava roots, unpeeled cassava roots, and cassava chips. There were no differences for phosphorus and sodium among the analyzed cassava co-products.

## DISCUSSION

The chemical composition of cassava co-products confirms that cassava is a high starch, low crude protein, and low AEE ingredient. The chemical composition of peeled cassava roots, unpeeled cassava roots, cassava chips, cassava meal, and cassava residue were generally within the range of published values for peeled cassava roots [12,13], non-peeled cassava roots [13,14], cassava chips [13,15,16], cassava meal [17–19], and cassava residue [13,20,21]. The analyzed components of unpeeled cassava roots, high-ash cassava meal, and cassava residue were close to 100%, indicating that all nutrients in these ingredients were accounted for [22]. However, despite extensive nutrient analysis of peeled cassava roots, cassava chips, and cassava meal, analyzed nutrients accounted only for 93.20% to 94.70% of these ingredients, and it is not clear what the remaining fractions consist of. It is possible that cyanogenic glucosides are present, but their concentration would be very low due to the processing used in the production of all samples [23]. It is, therefore, most likely that the rest fraction in these ingredients consists of resistant starch fractions that were not included in the starch analyzed via the glucoamylase procedure.

Fresh cassava roots typically contain 60% moisture and are primarily used to produce cassava starch, dried cassava (also known as chips), and animal feed pellets [23]. Sun-drying cleaned cassava roots usually takes 3 to 6 days, and steam can be used to speed up the process [23]. Mechanical raking is used to turn the chips throughout the drying process [23]. Following this process, cassava chips can be ground and pelleted, which greatly reduces the risk of toxicity to animals [23]. Cassava waste is also an important environmental concern, and waste can be classified into five types: starch, peels, stumps, whey, and effluent [7]. Cassava starch residue is a type of residue that is the fibrous and starch components left after the starch in the tubers has been extracted using screen separators [24]. Cassava-based ingredients usually provide energy to diets for livestock at a lower cost than cereal grains, particularly in developing countries [7,24].

Cassava co-products are considered sources of energy and can be used to replace cereal grains in diets, especially for pigs and poultry [5]. However, the main limitation of using cassava co-products for these animals is the low protein concentration and the lack of indispensable AA including the sulfur-containing AA [25]. In addition, cassava contains no pigment for yolk or skin coloring in poultry, therefore, synthetic pigments need to be added if cassava is included in poultry diets [23]. Diets with high levels of cassava need to be pelletized for non-ruminants, particularly for poultry, because powdered starch and dustiness from cassava have ulcerogenic effects on the gastric mucosa and can reduce feed intake [26]. Vegetable oils can also be used to improve energy digestibility and palatability of diets [27].

Cassava starch is more digestible than corn starch due to its high amylopectin content [23], but as demonstrated in this study, the contribution of AA and AEE from cassava products is very low. However, cassava can replace corn in animal diets if the hydrocyanic acid content does not exceed 100 mg/kg in the final diet [6]. If cassava is used as a major source of energy in diets, increased inclusion of soybean meal or another AA source is required [23,27].

Dried peeled cassava roots were expected to contain less ash and total dietary fiber than dried unpeeled cassava roots. However, this was not the case, and the lack of differences between these two ingredients indicates that removal of the peel has little impact on the composition of the end products, which may be because peel is only a minor part of the total ingredient, accounting for 5% to 15% of the tubers [28]. Peeling cassava roots for animal feeding is, therefore, not recommended because it has limited impact on chemical composition, which is likely why using unpeeled cassava usually results in satisfactory growth performance [23]. However, the washing process, which removes dirt, is important, and the low ash concentration in most cassava ingredients indicates that the washing process was effective in removing dirt from the tubers.

The observation that the composition of cassava meal and cassava chips is not different from that of peeled and unpeeled roots indicates that these cassava products are similar in composition. However, cassava meal contained slightly more ash and less starch than peeled and unpeeled cassava roots and cassava chips, which is consistent with previous data [13]. Cassava root meal has a varied profile due to differences in stage of harvesting, which can affect nutrient composition [13,29]. It is, however, possible that processes to produce cassava meal, cassava chips, peeled cassava roots, and unpeeled cassava roots are similar, but that different companies market the products under different names. Based on these observation, it appears to be of minor importance for feed manufactures if they buy one product or another.

The high-ash cassava meal from Indonesia was most likely produced from unwashed or improperly sifted roots. Due to the high Ca concentration that was analyzed, it is possible that limestone or sand were added to this product, and the nutritional value in terms of energy of the high-ash cassava meal is clearly less than that of the other co-products. Due to the high ash concentration, this ingredient likely needs to be used at low inclusion rates in diets for animals.

The low concentration of starch and the high concentration of total dietary fiber in cassava residue was expected because this is a co-product from the cassava starch industry. Nevertheless, because cassava residue contains 45% starch, it may be a valuable ingredient in diets for older pigs and ruminants. Cassava residue may also be used as silage for ruminants, but the quality of silage depends on plant age, time after harvest, industrial processing equipment, and processing method [30]. However, the fiber concentration in cassava residue may be too high for significant inclusion in diets for young pigs or poultry.

## CONCLUSION

Cassava co-products are excellent sources of energy, but are low in AEE and AA. If correctly managed, cassava can be used as a substitute for corn or other cereal grains in animal diets. Cassava-based co-products sold as peeled or unpeeled roots, chips, or meal generally have chemical compositions that are not different and peeling appears to have little impact on the chemical composition of cassava co-products. Because of the low starch and gross energy, high-ash cassava meal has a lower nutritional value compared with the other cassava products. Cassava residue may be used in diets for older pigs and ruminants, but the fiber concentration is likely too high for use in diets for younger pigs or poultry.

## Figures and Tables

**Table 1:** Analyzed nutrient composition of dried peeled cassava roots, as-fed basis

Item (%)	Philippines				Thailand

	Sample 1	Sample 2	CV	Average	
Dry matter	87.43	89.72	1.83	88.58	87.69
Gross energy (kcal/kg)	3,581	3,675	1.83	3,628	3,644
Crude protein	2.21	1.89	11.04	2.05	2.09
AEE	0.78	0.62	16.16	0.70	0.30
Ash	2.85	2.32	14.50	2.59	1.31
Carbohydrates					
Total starch	65.90	71.50	5.76	68.70	70.60
Insoluble dietary fiber	6.40	3.70	37.81	5.05	4.70
Soluble dietary fiber	0.70	1.30	42.43	1.00	1.40
Total dietary fiber	7.10	5.00	24.54	6.05	6.10
Glucose	0.47	0.43	6.29	0.45	0.12
Fructose	0.54	0.82	29.12	0.68	0.18
Maltose	0.06	ND	-	0.03	ND
Sucrose	0.73	0.13	98.67	0.43	0.26
Stachyose	ND	ND	-	-	ND
Raffinose	ND	ND	-	-	ND
Rest fraction^1)^	6.79	7.01	-	6.90	6.73
Indispensable AA					
Arginine	0.08	0.05	32.64	0.07	0.05
Histidine	0.03	0.02	28.28	0.03	0.02
Isoleucine	0.07	0.06	10.88	0.07	0.05
Leucine	0.10	0.08	15.71	0.09	0.06
Lysine	0.09	0.07	17.68	0.08	0.07
Methionine	0.02	0.01	47.14	0.02	0.01
Phenylalanine	0.06	0.05	12.86	0.06	0.05
Threonine	0.06	0.05	12.86	0.06	0.06
Tryptophan	0.03	0.03	0.00	0.03	0.03
Valine	0.08	0.06	20.20	0.07	0.05
Dispensable AA					
Alanine	0.11	0.11	0.00	0.11	0.09
Aspartic acid	0.14	0.13	5.24	0.14	0.10
Cysteine	0.01	0.01	0.00	0.01	0.01
Glutamic acid	0.30	0.25	12.86	0.28	0.29
Glycine	0.07	0.06	10.88	0.07	0.04
Proline	0.07	0.08	9.43	0.08	0.04
Serine	0.06	0.05	12.86	0.06	0.05
Tyrosine	0.03	0.02	28.28	0.03	0.03
Minerals					
Calcium	0.18	0.12	28.28	0.15	0.04
Phosphorus	0.15	0.12	15.71	0.14	0.04
Magnesium	0.10	0.06	35.36	0.08	0.02
Potassium	0.74	0.90	13.80	0.82	0.16
Sodium	0.01	0.01	0.00	0.01	0.01
Sulfur	0.05	0.04	15.71	0.05	0.01
Cooper (mg/kg)	4.05	4.31	4.40	4.18	3.44
Iron (mg/kg)	146.69	114.38	17.50	130.54	17.13
Manganese (mg/kg)	9.89	6.80	26.18	8.35	6.98
Zinc (mg/kg)	13.72	8.11	36.34	10.92	22.88

CV, coefficient of variation; AA, amino acids; AEE, acid-hydrolyzed ether extract; ND, not detected.

1) Rest fraction = calculated using the following equation: [Dry matter − (crude protein + AEE + ash + total dietary fiber + total starch + glucose + fructose + maltose + sucrose + stachyose + raffinose)].

**Table 2:** Analyzed nutrient composition of dried unpeeled cassava roots, as-fed basis

Item (%)	Philippines				Vietnam

	Sample 1	Sample 2	CV	Average	
Dry matter	87.35	88.08	0.59	87.72	88.42
Gross energy (kcal/kg)	3,607	3,635	0.55	3,621	3,616
Crude protein	2.19	2.02	5.71	2.11	2.31
AEE	0.71	0.78	6.64	0.75	0.86
Ash	2.42	3.64	28.47	3.03	1.84
Carbohydrates					
Total starch	67.00	65.90	1.17	66.45	70.50
Insoluble dietary fiber	8.10	8.40	2.57	8.25	6.30
Soluble dietary fiber	2.90	1.20	58.64	2.05	2.70
Total dietary fiber	11.00	9.60	9.61	10.30	9.00
Glucose	0.37	0.47	16.84	0.42	0.62
Fructose	0.07	0.06	10.88	0.07	0.76
Maltose	0.11	ND	-	0.06	0.76
Sucrose	ND	ND	-	-	0.85
Stachyose	ND	ND	-	-	-
Raffinose	ND	ND	-	-	-
Rest fraction^1)^	3.48	5.61	-	4.55	0.92
Indispensable AA					
Arginine	0.06	0.06	0.00	0.06	0.11
Histidine	0.03	0.03	0.00	0.03	0.02
Isoleucine	0.08	0.09	8.32	0.09	0.06
Leucine	0.11	0.13	11.79	0.12	0.09
Lysine	0.09	0.09	0.00	0.09	0.07
Methionine	0.02	0.03	28.28	0.03	0.02
Phenylalanine	0.07	0.08	9.43	0.08	0.06
Threonine	0.07	0.07	0.00	0.07	0.05
Tryptophan	0.02	0.02	0.00	0.02	0.03
Valine	0.09	0.09	0.00	0.09	0.07
Dispensable AA					
Alanine	0.10	0.10	0.00	0.10	0.11
Aspartic acid	0.14	0.15	4.88	0.15	0.11
Cysteine	0.02	0.02	0.00	0.02	0.02
Glutamic acid	0.28	0.20	23.57	0.24	0.25
Glycine	0.08	0.08	0.00	0.08	0.07
Proline	0.07	0.08	9.43	0.08	0.06
Serine	0.07	0.08	9.43	0.08	0.05
Tyrosine	0.03	0.03	0.00	0.03	0.03
Minerals					
Calcium	0.03	0.16	96.76	0.10	0.14
Phosphorus	0.03	0.15	94.28	0.09	0.09
Magnesium	0.03	0.07	56.57	0.05	0.10
Potassium	0.13	0.18	22.81	0.16	0.66
Sodium	0.01	0.02	47.14	0.02	0.01
Sulfur	0.01	0.04	84.85	0.03	0.03
Cooper (mg/kg)	5.23	5.35	1.60	5.29	5.09
Iron (mg/kg)	149.67	157.56	3.63	153.62	65.86
Manganese (mg/kg)	8.56	9.39	6.54	8.98	8.11
Zinc (mg/kg)	2.91	12.23	87.06	7.57	18.07

CV, coefficient of variation; AA, amino acids; AEE, acid-hydrolyzed ether extract; ND, not detected.

1) Rest fraction = calculated using the following equation: [Dry matter − (crude protein + AEE + ash + total dietary fiber + total starch + glucose + fructose + maltose + sucrose + stachyose + raffinose)].

**Table 3:** Analyzed nutrient composition of cassava chips, as-fed basis

Item (%)	Thailand	Vietnam						

		Sample 1	Sample 2	Sample 3	Sample 4	Sample 5	CV	Average
Dry matter	88.39	88.54	87.99	88.26	88.14	88.66	0.31	88.32
Gross energy (kcal/kg)	3,669	3,616	3,575	3,644	3,650	3,617	0.82	3,620
Crude protein	2.69	2.42	1.97	1.87	3.07	2.39	20.23	2.34
AEE	0.83	0.89	0.46	0.64	0.63	0.67	23.33	0.66
Ash	1.92	2.56	1.86	1.88	1.76	2.32	16.66	2.08
Carbohydrates								
Total starch	66.60	67.90	66.70	65.30	63.40	67.40	2.75	66.14
Insoluble dietary fiber	6.70	5.00	7.40	5.30	8.00	7.30	20.52	6.60
Soluble dietary fiber	1.80	2.70	0.30	1.10	1.10	2.80	68.75	1.60
Total dietary fiber	8.50	7.70	7.70	6.40	9.10	10.10	17.42	8.20
Glucose	0.19	0.76	0.43	1.09	0.71	0.45	39.17	0.69
Fructose	0.64	1.13	0.45	1.2	0.82	0.6	38.75	0.84
Maltose	ND	0.08	0.06	0.12	0.08	0	64.44	0.07
Sucrose	0.39	1.16	1.05	3.18	2.74	0.78	61.47	1.78
Stachyose	ND	ND	ND	ND	ND	ND	-	-
Raffinose	ND	ND	0.10	ND	0.07	ND	-	0.03
Rest fraction^1)^	6.63	3.94	7.21	6.58	5.76	3.95	-	5.49
Indispensable AA								
Arginine	0.12	0.09	0.12	0.07	0.31	0.13	66.54	0.14
Histidine	0.03	0.04	0.02	0.03	0.04	0.03	26.15	0.03
Isoleucine	0.07	0.07	0.06	0.06	0.06	0.07	8.56	0.06
Leucine	0.09	0.10	0.08	0.09	0.09	0.11	12.13	0.09
Lysine	0.09	0.09	0.07	0.08	0.09	0.09	10.65	0.08
Methionine	0.02	0.02	0.01	0.02	0.02	0.02	24.85	0.02
Phenylalanine	0.07	0.07	0.05	0.06	0.06	0.07	13.49	0.06
Threonine	0.07	0.07	0.05	0.06	0.06	0.06	11.79	0.06
Tryptophan	0.03	0.03	0.03	0.03	0.03	0.03	0.00	0.03
Valine	0.07	0.07	0.06	0.06	0.07	0.08	12.30	0.07
Dispensable AA								
Alanine	0.13	0.11	0.09	0.11	0.13	0.12	13.24	0.11
Aspartic acid	0.14	0.14	0.11	0.11	0.13	0.15	13.98	0.13
Cysteine	0.02	0.02	0.01	0.01	0.02	0.02	34.23	0.02
Glutamic acid	0.34	0.32	0.24	0.26	0.50	0.32	31.27	0.33
Glycine	0.07	0.07	0.06	0.06	0.07	0.08	12.30	0.07
Proline	0.06	0.08	0.06	0.06	0.07	0.07	12.30	0.07
Serine	0.07	0.07	0.05	0.05	0.06	0.07	16.67	0.06
Tyrosine	0.03	0.02	0.03	0.03	0.04	0.03	23.57	0.03
Minerals								
Calcium	0.09	0.11	0.05	0.08	0.11	0.12	30.65	0.09
Phosphorus	0.06	0.12	0.02	0.09	0.07	0.09	47.45	0.08
Magnesium	0.06	0.09	0.02	0.08	0.11	0.06	47.51	0.07
Potassium	0.38	0.62	0.17	0.45	0.50	0.76	43.98	0.50
Sodium	ND	0.02	ND	0.02	0.01	ND	100.00	0.01
Sulfur	0.03	0.04	0.01	0.04	0.04	0.04	39.46	0.03
Cooper (mg/kg)	2.20	3.03	1.78	2.56	3.36	8.70	70.92	3.89
Iron (mg/kg)	45.80	200.83	13.25	163.87	152.27	107.37	56.51	127.52
Manganese (mg/kg)	11.34	11.80	3.87	9.99	17.45	21.55	52.83	12.93
Zinc (mg/kg)	5.31	13.24	4.58	11.82	13.92	19.97	43.35	12.71

CV, coefficient of variation; AA, amino acids; AEE, acid-hydrolyzed ether extract; ND, not detected.

1) Rest fraction = calculated using the following equation: [Dry matter − (crude protein + AEE + ash + total dietary fiber + total starch + glucose + fructose + maltose + sucrose + stachyose + raffinose)].

**Table 4:** Analyzed nutrient composition of cassava meal, as-fed basis

Item (%)	Thailand			

	Sample 1	Sample 2	CV	Average
Dry matter	88.11	89.13	0.81	88.62
Gross energy (kcal/kg)	3,590	3,576	0.28	3,583
Crude protein	2.65	2.66	0.27	2.66
AEE	0.63	0.99	31.43	0.81
Ash	4.15	5.54	20.29	4.85
Carbohydrates				
Total starch	62.30	57.20	6.04	59.75
Insoluble dietary fiber	9.30	12.50	20.76	10.90
Soluble dietary fiber	1.80	3.60	47.14	2.70
Total dietary fiber	11.10	16.10	26.00	13.60
Glucose	0.53	0.61	9.92	0.57
Fructose	0.73	0.48	29.22	0.61
Maltose	0.09	0.06	28.28	0.08
Sucrose	0.64	0.13	93.67	0.39
Stachyose	ND	ND	-	-
Raffinose	ND	ND	-	-
Rest fraction^1)^	5.29	5.36	-	5.33
Indispensable AA				
Arginine	0.13	0.07	42.43	0.10
Histidine	0.04	0.03	20.20	0.04
Isoleucine	0.09	0.09	0.00	0.09
Leucine	0.13	0.12	5.66	0.13
Lysine	0.10	0.11	6.73	0.11
Methionine	0.02	0.02	0.00	0.02
Phenylalanine	0.08	0.08	0.00	0.08
Threonine	0.08	0.08	0.00	0.08
Tryptophan	0.03	0.03	0.00	0.03
Valine	0.10	0.10	0.00	0.10
Dispensable AA				
Alanine	0.13	0.14	5.24	0.14
Aspartic acid	0.17	0.17	0.00	0.17
Cysteine	0.02	0.02	0.00	0.02
Glutamic acid	0.36	0.28	17.68	0.32
Glycine	0.09	0.09	0.00	0.09
Proline	0.09	0.09	0.00	0.09
Serine	0.08	0.08	0.00	0.08
Tyrosine	0.04	0.03	20.20	0.04
Minerals				
Calcium	0.19	0.36	43.71	0.28
Phosphorus	0.08	0.07	9.43	0.08
Magnesium	0.09	0.09	0.00	0.09
Potassium	0.74	0.43	37.47	0.59
Sodium	0.02	0.04	47.14	0.03
Sulfur	0.04	0.05	15.71	0.05
Cooper (mg/kg)	6.44	6.26	2.00	6.35
Iron (mg/kg)	944.17	612.73	30.11	778.45
Manganese (mg/kg)	35.55	60.29	36.51	47.92
Zinc (mg/kg)	14.63	8.57	36.94	11.60

CV, coefficient of variation; AA, amino acids; AEE, acid-hydrolyzed ether extract; ND, not detected.

1) Rest fraction = calculated using the following equation: [Dry matter − (crude protein + AEE + ash + total dietary fiber + total starch + glucose + fructose + maltose + sucrose + stachyose + raffinose)].

**Table 5:** Analyzed nutrient composition of high-ash cassava meal, as-fed basis

Item (%)	Indonesia			

	Sample 1	Sample 2	CV	Average
Dry matter	92.64	91.84	0.61	92.24
Gross energy (kcal/kg)	2,135	2,147	0.40	2,141
Crude protein	1.78	1.96	6.81	1.87
AEE	0.49	0.67	21.94	0.58
Ash	38.66	35.68	5.67	37.17
Carbohydrates				
Total starch	28.80	30.30	3.59	29.55
Insoluble dietary fiber	21.20	21.50	0.99	21.35
Soluble dietary fiber	1.60	0.90	39.60	1.25
Total dietary fiber	22.80	22.40	1.25	22.60
Rest fraction^1)^	0.11	0.83	-	0.47
Indispensable AA				
Arginine	0.05	0.05	0.00	0.05
Histidine	0.03	0.03	0.00	0.03
Isoleucine	0.06	0.07	10.88	0.07
Leucine	0.10	0.11	6.73	0.11
Lysine	0.08	0.09	8.32	0.09
Methionine	0.02	0.03	28.28	0.03
Phenylalanine	0.06	0.06	0.00	0.06
Threonine	0.06	0.07	10.88	0.07
Tryptophan	0.02	0.02	0.00	0.02
Valine	0.08	0.08	0.00	0.08
Dispensable AA				
Alanine	0.08	0.08	0.00	0.08
Aspartic acid	0.11	0.13	11.79	0.12
Cysteine	0.01	0.02	47.14	0.02
Glutamic acid	0.13	0.14	5.24	0.14
Glycine	0.06	0.08	20.20	0.07
Proline	0.07	0.07	0.00	0.07
Serine	0.06	0.09	28.28	0.08
Tyrosine	0.03	0.04	20.20	0.04
Minerals				
Calcium	9.56	8.06	12.04	8.81
Phosphorus	0.11	0.09	14.14	0.10
Magnesium	4.19	1.64	61.86	2.92
Potassium	7.29	5.06	25.54	6.18
Sodium	0.03	0.03	0.00	0.03
Sulfur	0.56	0.70	15.71	0.63
Cooper (mg/kg)	86.95	74.00	11.38	80.48
Iron (mg/kg)	2622.17	2320.77	8.62	2471.47
Manganese (mg/kg)	487.22	576.83	11.91	532.03
Zinc (mg/kg)	207.02	311.29	28.45	259.16

CV, coefficient of variation; AA, amino acids; AEE, acid-hydrolyzed ether extract.

1) Rest fraction = calculated using the following equation: [Dry matter − (crude protein + AEE + ash + total dietary fiber + total starch)].

**Table 6:** Analyzed nutrient composition of cassava residue, as-fed basis

Item (%)	Indonesia	Thailand					Vietnam			
	
		Sample 1	Sample 2	Sample 3	CV	Average	Sample 1	Sample 2	CV	Average
Dry matter	88.19	87.28	87.63	87.31	0.22	87.41	89.33	89.66	0.26	89.50
Gross energy (kcal/kg)	3,637	3,542	3,515	3,491	0.73	3,516	3,660	3,665	0.10	3,663
Crude protein	2.52	2.50	2.71	2.03	14.43	2.41	1.96	2.15	6.54	2.06
AEE	1.98	0.91	1.65	0.62	50.11	1.06	1.26	0.83	29.10	1.05
Ash	2.81	4.22	5.18	5.33	12.27	4.91	1.74	1.29	21.00	1.52
Carbohydrates										
Total starch	45.80	44.60	36.20	49.20	15.21	43.33	46.60	50.00	4.98	48.30
Insoluble dietary fiber	28.20	28.70	34.80	24.40	17.84	29.30	36.60	29.40	15.43	33.00
Soluble dietary fiber	5.50	3.70	5.20	4.50	16.80	4.47	4.10	4.70	9.64	4.40
Total dietary fiber	33.70	32.40	40.00	28.90	16.81	33.77	40.70	34.10	12.48	37.40
Rest fraction^1)^	1.38	2.65	1.89	1.23	-	1.92	−2.93	1.29	-	−0.82
Indispensable AA										
Arginine	0.06	0.06	0.07	0.05	16.67	0.06	0.17	0.17	0.00	0.17
Histidine	0.04	0.04	0.04	0.03	15.75	0.04	0.04	0.03	20.20	0.04
Isoleucine	0.08	0.09	0.10	0.08	11.11	0.09	0.06	0.05	12.86	0.06
Leucine	0.12	0.14	0.15	0.11	15.61	0.13	0.09	0.07	17.68	0.08
Lysine	0.13	0.13	0.17	0.11	22.35	0.14	0.12	0.09	20.20	0.11
Methionine	0.03	0.03	0.03	0.02	21.65	0.03	0.02	0.01	47.14	0.02
Phenylalanine	0.07	0.08	0.08	0.06	15.75	0.07	0.05	0.05	0.00	0.05
Threonine	0.08	0.09	0.09	0.07	13.86	0.08	0.05	0.04	15.71	0.05
Tryptophan	0.02	0.02	0.02	0.02	0.00	0.02	0.02	0.02	0.00	0.02
Valine	0.10	0.11	0.12	0.09	14.32	0.11	0.07	0.06	10.88	0.07
Dispensable AA										
Alanine	0.10	0.12	0.14	0.10	16.67	0.12	0.06	0.05	12.86	0.06
Aspartic acid	0.14	0.17	0.19	0.13	18.70	0.16	0.11	0.09	14.14	0.10
Cysteine	0.02	0.03	0.02	0.01	50.00	0.02	0.02	0.01	47.14	0.02
Glutamic acid	0.16	0.19	0.21	0.15	16.66	0.18	0.13	0.12	5.66	0.13
Glycine	0.08	0.09	0.10	0.07	17.63	0.09	0.05	0.04	15.71	0.05
Proline	0.09	0.09	0.10	0.07	17.63	0.09	0.08	0.06	20.20	0.07
Serine	0.08	0.09	0.09	0.07	13.86	0.08	0.07	0.05	23.57	0.06
Tyrosine	0.03	0.04	0.04	0.03	15.75	0.04	0.04	0.02	47.14	0.03
Minerals										
Calcium	0.34	0.74	1.30	1.26	28.40	1.10	0.32	0.26	14.63	0.29
Phosphorus	0.03	0.04	0.08	0.04	43.30	0.05	0.02	0.03	28.28	0.03
Magnesium	0.09	0.13	0.22	0.11	38.21	0.15	0.12	0.08	28.28	0.10
Potassium	0.25	0.29	0.59	0.31	42.28	0.40	0.34	0.29	11.22	0.32
Sodium	0.03	0.05	0.06	0.13	54.49	0.08	0.01	0.01	0.00	0.01
Sulfur	0.05	0.05	0.08	0.06	24.12	0.06	0.06	0.05	12.86	0.06
Cooper (mg/kg)	3.99	5.05	7.02	4.99	20.31	5.69	11.64	4.85	58.23	8.25
Iron (mg/kg)	892.04	674.83	496.23	201.85	52.19	457.64	126.34	89.70	23.98	108.02
Manganese (mg/kg)	42.31	41.19	53.95	33.94	23.55	43.03	28.44	16.12	39.10	22.28
Zinc (mg/kg)	10.14	10.66	19.10	9.56	39.82	13.11	8.58	7.38	10.63	7.98

CV, coefficient of variation; AA, amino acids; AEE, acid-hydrolyzed ether extract.

1) Rest fraction = calculated using the following equation: [Dry matter − (crude protein + AEE + ash + total dietary fiber + total starch)].

**Table 7:** Comparison of chemical composition of cassava-based feed ingredients, as-fed basis

Item (%)	Peeled cassava roots	Unpeeled cassava roots	Cassava chips	Cassava meal	High-ash cassava meal	Cassava residue	SEM	p-value
Dry matter	88.28^b^	87.95^b^	88.33^b^	88.62^b^	92.24^a^	88.23^b^	0.45	0.044
Gross energy (kcal/kg)	3,633^a^	3,619^a^	3,629^a^	3,583^b^	2,141^c^	3,585^ab^	18.76	<0.001
Crude protein	2.06	2.17	2.40	2.66	1.87	2.31	0.18	0.170
AEE	0.57	0.78	0.69	0.81	0.58	1.21	0.19	0.066
Ash	2.16^c^	2.63^bc^	2.05^c^	4.85^b^	37.17^a^	3.43^bc^	0.71	<0.001
Carbohydrates								
Total starch	69.33^a^	67.80^a^	66.22^a^	59.75^b^	29.55^d^	45.40^c^	1.93	<0.001
Insoluble dietary fiber	4.94^d^	7.61^cd^	6.62^cd^	11.17^c^	21.62^b^	30.35^a^	1.72	<0.001
Soluble dietary fiber	1.13^b^	2.27^b^	1.63^b^	2.70^b^	1.25^b^	4.62^a^	0.48	<0.001
Total dietary fiber	6.07^d^	9.87^bc^	8.25^c^	13.60^b^	22.60^a^	34.97^a^	1.64	<0.001
Glucose	0.34	0.49	0.61	0.57	-	-	0.17	0.585
Fructose	0.51	0.30	0.81	0.61	-	-	0.18	0.207
Maltose	0.02	0.29	0.06	0.08	-	-	0.11	0.320
Sucrose	0.37	0.28	1.55	0.39	-	-	0.49	0.138
Stachyose	-	-	-	-	-	-	-	-
Raffinose	-	-	0.03	-	-	-	0.02	0.470
Rest fraction^1)^	6.84^a^	3.34^bc^	5.68^ab^	5.33^ab^	0.47^cd^	0.92^d^	0.92	0.0002
Indispensable AA								
Arginine	0.09	0.10	0.14	0.13	0.08	0.10	0.03	0.173
Histidine	0.02	0.03	0.03	0.03	0.03	0.04	0.00	0.062
Isoleucine	0.06	0.08	0.07	0.09	0.06	0.08	0.01	0.177
Leucine	0.08	0.11	0.09	0.13	0.11	0.11	0.01	0.160
Lysine	0.08^b^	0.08^b^	0.09^b^	0.11^ab^	0.09^b^	0.13^a^	0.01	0.004
Methionine	0.01	0.02	0.02	0.02	0.03	0.02	0.00	0.223
Phenylalanine	0.05	0.07	0.06	0.08	0.06	0.07	0.01	0.137
Threonine	0.05	0.06	0.06	0.08	0.06	0.07	0.01	0.371
Tryptophan	0.03^a^	0.02^b^	0.03^a^	0.03^a^	0.02^bc^	0.02^c^	0.00	<0.001
Valine	0.06	0.08	0.07	0.10	0.08	0.09	0.01	0.050
Dispensable AA								
Alanine	0.10	0.10	0.12	0.14	0.08	0.10	0.01	0.165
Aspartic acid	0.12	0.13	0.13	0.17	0.12	0.14	0.01	0.396
Cysteine	0.01	0.02	0.02	0.02	0.01	0.02	0.00	0.136
Glutamic acid	0.28^a^	0.24^ab^	0.33^a^	0.32^a^	0.14^b^	0.16^b^	0.03	0.001
Glycine	0.06	0.08	0.07	0.09	0.07	0.07	0.01	0.320
Proline	0.06	0.07	0.07	0.09	0.07	0.08	0.01	0.101
Serine	0.05	0.07	0.06	0.08	0.07	0.08	0.01	0.155
Tyrosine	0.03	0.03	0.03	0.04	0.04	0.03	0.00	0.613
Minerals								
Calcium	0.11^c^	0.11^c^	0.09^c^	0.28^bc^	8.81^a^	0.70^b^	0.22	<0.001
Phosphorus	0.10	0.09	0.08	0.08	0.10	0.04	0.02	0.191
Magnesium	0.06^b^	0.07^b^	0.07^b^	0.09^b^	2.92^a^	0.13^b^	0.26	<0.001
Potassium	0.60^b^	0.62^b^	0.48^b^	0.59^b^	6.18^a^	0.35^b^	0.27	<0.001
Sodium	0.01	0.01	0.01	0.03	0.03	0.05	0.02	0.070
Sulfur	0.03^b^	0.03^b^	0.03^b^	0.04^b^	0.63^a^	0.06^b^	0.02	<0.001
Cooper (mg/kg)	3.93^b^	5.22^b^	3.61^b^	6.35^b^	80.48^a^	6.26^b^	1.81	<0.001
Iron (mg/kg)	92.73^d^	124.36^cd^	113.90^d^	726.17^b^	2,419.19^a^	413.50^bc^	125.21	0.004
Manganese (mg/kg)	7.71^d^	8.50^cd^	12.67^d^	47.76^b^	531.86^a^	35.99^bc^	10.82	<0.001
Zinc (mg/kg)	14.93^b^	11.09^b^	11.47^b^	11.76^b^	259.31^a^	10.90^b^	11.39	<0.001

SEM, standard error of the means; AA, amino acids; AEE, acid-hydrolyzed ether extract.

1) Rest fraction = calculated using the following equation: [Dry matter − (crude protein + AEE + ash + total dietary fiber + total starch + glucose + fructose + maltose + sucrose + stachyose + raffinose)]. The rest fraction for high-ash cassava meal and cassava residue was calculated using the same equation, with the exception that sugars were not analyzed in these samples.

a–d Means in a row without a common superscript letter differ (p<0.05).
